# Pattern of symptom improvement following endoscopic sinus surgery for chronic rhinosinusitis

**DOI:** 10.1186/s12893-021-01269-1

**Published:** 2021-06-08

**Authors:** Salma S. Al Sharhan, Mohammed H. Al Bar, Shahad Y. Assiri, Assayl R. AlOtiabi, Deemah M. Bin-Nooh, Fozia K. AlSugair, Nada F. AlRashidi, Abdulmalik S. AlSaied, Amal A. Alghamdi

**Affiliations:** 1 Department of Otorhinolaryngology, College of Medicine, Imam Abdulrahman Bin Faisal University, Al Khobar, Saudi Arabia; 2 Division of Epidemiology and Biostatistics, Department of Family and Community Medicine, College of Medicine, Imam Abdulrahman Bin Faisal University, Al Khobar, Saudi Arabia

**Keywords:** Chronic rhinosinusitis, Quality of life, Nasal polyps, Pattern of symptoms

## Abstract

**Background:**

Chronic rhinosinusitis (CRS) is a common inflammation of the nose and the paranasal sinuses. Intractable CRS cases are generally treated with endoscopic sinus surgery (ESS). Although the effect of ESS on CRS symptoms has been studied, the pattern of symptom improvement after ESS for CRS is yet to be investigated. The aim of this study was to determine the magnitude and sequence of symptom improvement after ESS for CRS, and to assess the possible preoperative factors that predict surgical outcomes in CRS patients.

**Methods:**

This was a longitudinal prospective study of 68 patients who had CRS (with or without nasal polyps). The patients underwent ESS at King Fahd Hospital of the University, Al Khobar, Saudi Arabia. The Sino-nasal Outcome Test-22 (SNOT-22) questionnaire was used for assessment at four time points during the study: pre-ESS, 1-week post-ESS, 4 weeks post-ESS, and 6 months post-ESS.

**Results:**

The difference between the mean scores recorded for the five SNOT-22 domains pre-ESS and 6 months post-ESS were as follows: rhinologic symptoms (t-test = 7.22, p-value =  < 0.001); extra-nasal rhinologic symptoms (t-test = 4.87, p-value =  < 0.001); ear/facial symptoms (t-test = 6.34, p-value =  < 0.001); psychological dysfunction (t-test = 1.99, p-value = 0.049); and sleep dysfunction (t-test = 5.58, p-value =  < 0.001). There was a significant difference between the mean scores recorded for the five domains pre-ESS and 6 months post-ESS. Rhinologic symptoms had the largest effect size (*d* = 1.12), whereas psychological dysfunction had the least effect size (*d* = 0.24). The only statistically significant difference in the SNOT-22 mean scores recorded 4 weeks post-ESS was observed between allergic and non-allergic patients (t = − 2.16, df = 66, p = 0.035).

**Conclusion:**

Understanding the pattern of symptom improvement following ESS for CRS will facilitate patient counselling and aid the optimization of the current treatment protocols to maximize surgical outcomes and quality of life.

**Level of evidence:**

Prospective observational.

## Background

Chronic rhinosinusitis (CRS) is an inflammation of the nose and the paranasal sinuses that is characterized by two or more of the following signs and symptoms, which persist for more than 12 consecutive weeks: nasal obstruction and/or congestion, nasal discharge (anterior and/or posterior), facial pain and/or pressure, and reduction or loss of smell. It is a common clinical condition that has a significant impact on quality of life and individual morbidity; CRS cases represent 10.8% of all outpatient clinical visits [1, 2]. Uncontrolled CRS symptoms may lead to impaired quality of life such as absence from work up to 6.5% of the time, a 38% loss of productivity, and a 36% reduction in on-the-job [3, 4]. The combination of different disorders results in complexes of symptoms, with each complex affecting quality of life differently [5].

Appropriate management of CRS is geared towards relieving symptoms and substantially improving the patient’s state of health; this can be achieved initially by pharmacological therapy [6]. However, in cases of recalcitrant CRS with no improvement of symptoms, endoscopic sinus surgery (ESS) is indicated [6, 7]. Development of a universal and standard treatment for CRS has been elusive for decades [8]. ESS, which is a precise guided intervention, improves both the permeability and performance of the nasal sinus spaces. This results in the proper ventilation and drainage of the facial sinuses [9]. Although few studies have examined the effect of ESS on CRS symptoms and quality of life after surgery, no study has investigated the pattern of symptom improvement after ESS for CRS. Therefore, the aim of this study was to determine the magnitude and sequence of symptom improvement over time after ESS for CRS, and to highlight the possible preoperative factors that predict surgical outcomes in CRS patients.

## Methods

This study was approved by the institutional review board of Imam Abdulrahman bin Faisal University (IRB No. UGS-2018-01-312). A written consent was taken from participants before their enrolment in the study. This was a prospective cohort that was conducted over the first 6 months after ESS. We recruited patients who visited the specialized Rhinology Clinics of the Otolaryngology Department at King Fahad Hospital of University, AlKhobar, Saudi Arabia whom were diagnosed based on the diagnostic clinical criteria of EPOS 2020 for CRS to have either chronic rhinosinusitis with nasal polyposis (CRSwNP) or chronic rhinosinusitis without nasal polyposis (CRSsNP) [2]. The participants were included consecutively and only patients who had refractory CRS (despite undergoing medical treatment for at least 12 weeks) and underwent bilateral ESS from August 2017 to September 2019, they were followed up for 6 months post. The detailed history of each participant was obtained and nasal endoscopic examination was performed on each participant, followed by confirmatory computed tomography scans, which were scored using the Lund-Mackay scoring system [10]. Any patient with underlying diseases such as malignancy, autoimmune diseases, granulomatous disease, cystic fibrosis, which may affect the management outcomes, were excluded.

All ESS procedures were conducted by an expert surgeon, who used a standard technique in an optimal setting. A unified medication protocol, which included hypertonic saline nasal irrigation and intranasal administration of corticosteroids and antibiotics, was prescribed for all patients.

Sociodemographic data were extracted from each participant’s medical records. The extracted data included: sex (male vs female), age, smoking history (smoker vs non-smoker), history of alcohol consumption (drinks alcohol vs does not drink alcohol), and presence of comorbidities (diabetes mellitus, hypertension, and laryngopharyngeal reflux disease). In addition, data on predictive factors including history of previous ESS, endoscopic presence of polyps, history of bronchial asthma, and allergic rhinitis (AR), were extracted as well.

All patients were routinely examined the 1st week, the 4th week, and around 6 months after surgery. During each visit, each participant had to complete the Arabic version of the Sino-nasal Outcome Test-22 (SNOT-22) questionnaire “All rights reserved. Copyright 2006. Washington University in St. Louis, Missouri.”, which is a disease-specific health-related quality of life assessment tool used for assessing symptoms of CRS with or without nasal polyps [10, 11]. The SNOT-22 questionnaire consists of 22 items that reflect the health burden of CRS symptoms. The items are categorized into five main domains: rhinological symptoms domain, extra-nasal rhinological symptoms domain, ear and facial symptoms domain, sleep dysfunction domain, and psychological disease domain. The rhinological symptoms domain consists of the following items: need to blow the nose, sneezing, post-nasal discharge, and nasal blockage. The extra-nasal rhinological domain consists of the following items: runny nose, cough, and post-nasal discharge. The ear and facial domain consists of the following items: ear fullness, dizziness, and ear pain. The sleep dysfunction domain consists of the following items: difficulty falling asleep, waking up at night, and lack of a good night sleep. Finally, the psychological domain consists of the following items: fatigue, reduced productivity, reduced concentration, and feeling frustrated/restless/irritable [12]. Each item is scored from 0 to 5, with 0 representing no problem and 5 representing the highest level of the problem.

Mean and standard deviation were used to describe and compare the initial and follow up values of the additive global score of SNOT-22 questionnaire as well as the additive scores of the five SNOT-22 subscale, whereas median was used to describe the pattern of the initial and follow up values of Likert scale of the 22 items of SNOT-22 questionnaire. Cohen’s d effect size was used to estimate the magnitude of change in the rhinologic symptoms over the 6-month period. The effect size was calculated by subtracting the mean SNOT-22 score recorded at the 6-month timepoint from the mean SNOT-22 score recorded prior to the surgery, before dividing it by the standard deviation of the mean difference. An effect size can be mild (0.4), moderate (0.5), or large (0.8). The two-sample t-test was used as appropriate to investigate the individual factors that may affect the SNOT-22 score at the 6-month time point, whereas the paired t-test and Wilcoxon sign rank test were used as appropriate to investigate the difference between the SNOT-22 global scores, subscales, and items recorded prior to the surgery and those recorded at the end of the follow-up period. Additionally, multiple linear regression models were run to estimate the magnitude of change in the SNOT22 score at the end of the study period in relation to various possible predicators. Finally, the line graph was used to display the trend of each measured SNOT-22 item over several time points within the follow-up period. A p value below 0.05 was considered statistically significant [13]. All statistical analyses were performed using SPSS version 24, and the line graph was drawn using Microsoft Office 350 excel [12].

## Results

A total of 110 participants met the inclusion criteria and were eligible to be enrolled in the study. However, due to loss of follow-up only 68 patients (61.82%) completed the follow-up questionnaires for the entire study period (i.e. 6 months).

Regarding the sociodemographic features of the participants, the mean age of the participants was 36.58 years (SD = 14.09, minimum = 13, maximum = 69) years. In addition, as shown in Table 1, from the included participants 54.41% (n = 37) were male, 47.06% (n = 32) underwent ESS for the first time, whiles 52.94% (n = 36) had ESS as a revision surgery. Moreover, 11.76% (n = 8) of the participants were smokers, and only one participant had a history of alcohol consumption.

**Table 1:** The distribution of the study participants’ characteristics

Variable	Number (n = 68)	Percent (100%)
Age (years)		
< 19	5	7.35
20–29	20	29.41
30–39	15	22.06
40–49	14	20.59
≥ 50	14	20.59
Gender		
Male	37	54.41
Female	31	45.59
Smoking		
No	60	88.24
Yes	8	11.76
Alcohol		
No	67	67
Yes	1	1
Diabetes mellitus		
No	65	95.59
Yes	3	4.41
Hypertension		
No	65	95.59
Yes	3	4.41
Laryngopharyngeal reflux disease		
No	63	92.65
Yes	5	7.35
Asthma		
No	53	77.94
Yes	15	22.06
Allergy		
No	30	44.12
Yes	38	55.88
Polyps		
No	48	70.59
Yes	20	29.41
ESS status		
First time surgery	32	47.06
Revision surgery	36	52.94

In addition, the diagnosis of bronchial asthma was confirmed in 22% (n = 15) of the participants, 29.4% (n = 20) of the participants had CRSwNP, and positive history of AR was reported in 55.88% (n = 38) of the participants (Table 1).

As seen in Table 2, the standard deviations of the five SNOT-22 domains showed a significant difference between the mean recorded pre-ESS and that recorded 6 months post-ESS. The largest effect size is seen in the rhinological symptoms domain (*d* = 1.12) and the least was noted the psychological dysfunction domain (*d* = 0.24).

**Table 2:** The mean, standard deviation and the Cohen’s d effect size of the subscales of the main five domains of the SNOT-22 questionnaire over the study period

SNOT-22 (Five main domains)	SNOT-22 domains’ scores over time												Difference in the score means between preoperative and average of 6 months post ESS					
	Preoperative			1-week post ESS			1-month post ESS			Average of 6 months post ESS			Significance of the differeNce			Raw mean difference	d	Magnitude
	M	SD	Mdn	M	SD	Mdn	M	SD	Mdn	M	SD	Mdn	t	df	P			
Rhinologic symptoms (0–30)	18.66	3.78	19.5	12.47	5.16	12.5	11.56	8.03	13.5	9.12	8.15	9.00	**7.22**	**67**	**< 0.001**	**− **9.54	1.12	Large
Extra nasal rhino logic symptoms (0–15)	6.99	2.00	7	6.01	2.13	6	3.69	1.46	3.5	4.80	3.44	3.75	**4.87**	**67**	**< 0.001**	**− **2.19	0.59	Moderate
Ear/facial symptoms (0–25)	9.09	2.77	9	5.62	3.85	4.5	6.52	6.02	6.5	4.79	5.79	2	**6.34**	**67**	**< 0.001**	**− **4.29	0.77	Moderate
Psychological dysfunction (0–35)	6.41	6.43	4.5	3.46	4.39	2	7.75	8.66	4.5	4.24	6.52	0	**1.99**	**67**	**0.049**	− 2.18	0.24	Mild
Sleep dysfunction (0–25)	7.61	4.17	7	3.96	3.70	3	6.31	7.01	3.5	3.62	5.00	0	**5.58**	**67**	**< 0.001**	3.99	0.68	Moderate
SNOT-22 (0–110)	40.30	12.34	39	26	14.20	23	30.88	24.36	29.5	22.18	20.76	17.5	**6.75**	**67**	**< 0.001**	18.12	0.82	Large

(M) mean, (Mdn) median, (d) Standardized mean difference, (t) t test value, (p) p value significant results set at < 0.05, (df) degree of freedom

**Fig. 1:**
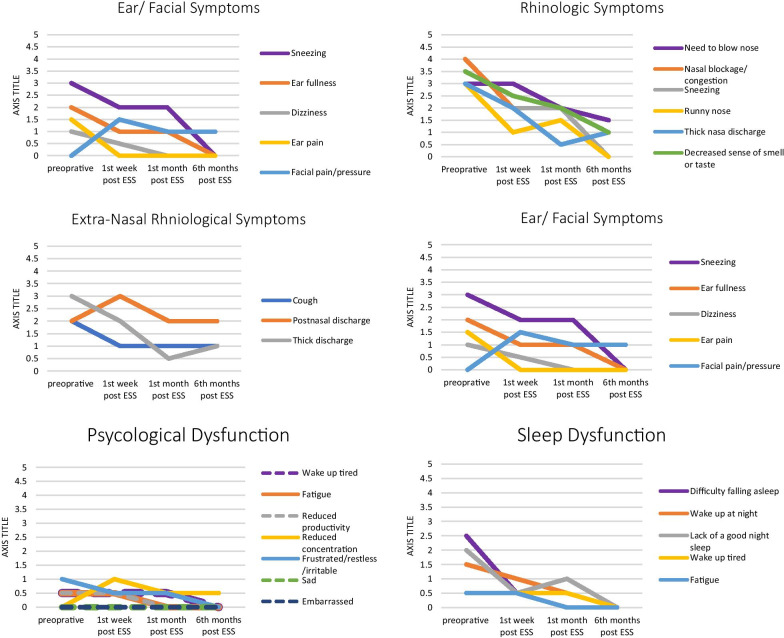
Line graph showing the trend of the rhinologic-related symptoms as measured with the five domains of the SNOT-22

As demonstrated in the line graph (Fig. 1), the symptoms either completely improved (i.e., the initial preoperative score dropped to 0 six months after surgery), partially improved (i.e., the initial preoperative score did not reach 0 six months after surgery) or showed no improvement (i.e., the initial preoperative score remained the same 6 months after surgery). The following symptoms had the highest median scores: need to blow nose, nasal blockage, sneezing, runny nose, and decreased sense of smell/taste. However, these symptoms significantly improved to a ‘very mild problem’ in the 6th month after surgery: need to blow nose (z = − 3.93, p =  < 0.001), nasal blockage (z = − 6.44, p =  < 0.001), sneezing (z = − 5.88, p =  < 0.001), runny nose (z = − 6.21, p =  < 0.001), and decreased sense of smell/taste (z = − 5.17, p =  < 0.001). On the other hand, the median score for post-nasal discharge, which is known bothersome symptom of CRS, did not change after surgery (z = − 0.27, p = 0.77). The results of Wilcoxon sign rank test display the difference between the medians of the SNOT-22 items before surgery and 6 months post-ESS (Table 3).

**Table 3:** The distribution of the SNOT-22 items over the four time points and the results of Wilcoxon sign rank test that shows the difference between the median of the SNOT-22 items preoperative and 6 months post ESS

Items	Preoperative			1st week post ESS			1st month post ESS			6th months post ESS			Wilcoxon sign rank	
	Mdn	M	SD	Mdn	M	SD	Mdn	M	SD	Mdn	M	SD	Z	p
Need to blow nose	3.00	2.91	0.94	3.00	2.81	1.14	2.00	2.06	0.91	1.50	1.91	1.65	**− 3.93**	**< 0.001**
Nasal blockage	4.00	3.08	0.94	2.00	2.04	1.02	2.00	2.03	0.77	1.00	1.53	1.065	**− 6.44**	**< 0.001**
Sneezing	3.00	2.84	0.70	2.00	2.00	0.93	2.00	1.99	0.82	0.00	1.19	1.65	**− 5.88**	**< 0.001**
Runny nose	3.00	2.87	0.86	1.00	1.19	1.08	1.50	1.57	0.83	0.00	1.06	1.43	**− 6.21**	**< 0.001**
Cough	2.00	2.00	0.86	1.00	1.03	0.67	1.00	1.00	0.62	1.00	1.03	1.16	**− 4.86**	**< 0.001**
Post-nasal discharge	2.00	2.10	0.88	3.00	2.93	0.92	2.00	2.00	0.69	2.00	2.10	2.10	**− **0.27	0.770
Thick discharge	3.00	2.88	0.94	2.00	2.06	1.06	0.50	0.69	0.90	1.00	1.70	1.78	**− 0.450**	**< 0.001**
Ear fullness	2.00	2.01	0.89	1.00	1.22	1.08	1.00	1.44	1.63	0.00	1.07	1.54	**− 4.18**	**< 0.001**
Dizziness	1.00	1.12	0.87	0.50	0.75	0.94	0.00	0.81	1.52	0.00	0.72	1.39	**− 3.22**	**0.001**
Ear pain	1.50	1.56	0.57	0.00	0.38	1.08	0.00	0.88	1.45	0.00	0.82	1.30	**− 4.09**	**< 0.001**
Facial pain/pressure	0.00	0.99	1.61	1.50	1.56	0.90	1.00	1.26	1.14	1.00	1.5	1.83	**− 2.89**	**0.003**
Decreased sense of smell/taste	3.50	3.35	1.09	2.50	2.37	1.19	2.00	2.13	1.89	1.00	1.74	1.87	**− 5.17**	**< 0.001**
Difficulty falling asleep	2.50	2.40	0.79	0.50	0.75	0.96	1.00	1.31	1.62	0.00	0.79	1.25	**− 6.18**	**< 0.001**
Wake up at night	1.50	1.59	1.01	1.00	1.06	0.77	0.50	1.13	1.45	0.00	0.59	1.01	**− 3.26**	**0.001**
Lack of a good night’s sleep	2.00	2.03	0.93	0.50	0.69	0.86	1.00	1.50	1.7	0.00	0.74	1.30	**− 5.57**	**< 0.001**
Wake up tired	0.50	0.74	0.91	0.50	0.72	0.93	0.50	1.31	1.66	0.00	0.69	1.18	1.14	0.260
Fatigue	0.50	0.86	1.10	0.50	0.74	0.93	0.00	1.06	1.57	0.00	0.81	1.31	**− **1.31	0.190
Reduced productivity	0.50	0.68	0.81	0.50	0.72	0.92	0.00	1.00	1.55	0.00	0.54	1.07	**− **1.70	0.089
Reduced concentration	0.00	0.62	1.06	1.00	1.15	0.85	0.50	0.72	0.93	0.50	1.19	1.52	**− 3.49**	**0.004**
Frustrated/restless/irritable	1.00	1.18	0.95	0.5	0.74	0.93	0.50	1.31	1.66	0.00	0.53	1.24	**− 4.20**	**< 0.001**
Sad	0.00	0.46	1.21	0.00	0.34	1.08	0.00	0.12	0.56	0.00	0.88	1.5	1.12	0.305
Embarrassed	0.00	0.34	1.13	0.00	0.12	0.56	0.00	1.00	1.51	0.00	0.59	1.24	1.82	0.074

As shown in Table 4, the only statistically significant difference in the SNOT-22 mean scores recorded 4 weeks after surgery was found between participants who had allergic rhinitis and non-allergic patients (t = − 2.16, df = 66, p = 0.035). Likewise to that previous finding, allergy was found to be the only statically significant predictor for SNOT-22 score at the 6 months period as it was found that having an allergy will increase the SNOT-22 score by 12 units after adjustment for gender, smoking and diabetes (β = 11.98, SE = 4.88, p = 0.017, F(4,63) = 0.036, adjusted R2 = 0.095, R2 = 0.149).

**Table 4:** The differences in the SNOT-22 score at 6 months period in relation to various risk factors

Predictive factors	6 months post EES SNOT-22 score				
	Mean	SD	t	df	p value
Age (years)			0.39*	4.63	0.816
< 19	23.6	33.75			
20–29	26.15	20.80			
30–39	18.53	15.99			
40–49	18.86	23.44			
≥ 50	23.25	18.98			
Sex			− 1.23	66	0.889
Male	19.35	3.38			
Female	25.56	3.74			
Asthma			− 0.91	66	0.366
Without	20.96	2.74			
With	26.50	6.09			
Smoking			− 1.07	66	0.288
Without	21.2	2.67			
With	29.56	7.61			
Previous sinus surgery			− 0.18	66	0.854
Without	21.69	3.46			
With	22.62	3.67			
Allergies			− **2.16**	**66**	**0.035**
Without	16.23	3.42			
With	26.88	3.46			
Polyps					
Without	23.47	2.96	− 0.79	66	0.433
With	19.10	4.83			
Culture positive			− 0.80	66	0.496
Positive	18.56	5.42			
Negative	23.30	2.85			
Medical comorbidities			− 0.29	66	0.77
Without	21.71	2.91			
With	23.33	5.06			

(t) = t-test statistics, (df) = degree of freedom, (p) = p-value of t test

## Discussion

In this study, we aimed to determine the effectiveness of ESS among CRS patients during the 6-months postoperative period before they are discharged from the clinic. By the end of this period, the nasal mucosa would have healed and the function of the sinuses would be regained. The symptoms of CRS can affect quality of life significantly. For cases in which medical treatment did not improve the symptoms, ESS is an alternative therapeutic solution for improving rhinological symptoms. Nevertheless, there have been controversies about its effectiveness [9, 10, 13].

After analyzing the magnitude and pattern of symptom improvement in all five domains of the SNOT-22 score system, we found a statistically significant reduction in the subscales of the five main domains, all of which had large to moderate effect sizes; the least magnitude was observed in the psychological dysfunction domain. Each of the involved symptoms showed varying degrees and patterns of improvement over 6 months.

In the 1st month, nasal blockage, sneezing, runny nose, thick nasal discharge, and a decreased sense of smell/taste showed a marked and dramatic improvement. This indicates that surgical management achieved patency of the nasal cavity and sinus drainage system, which were obstructed by the disease prior to the surgery. In addition, most of the symptoms, such as lack of a good night’s sleep and difficulty falling asleep, which represent the sleep dysfunction domain, were resolved and patients had less interrupted and disturbed sleep.

In the 6th month, some of the symptoms, such as nasal blockage, runny nose, sneezing thick nasal discharge, and dizziness, continued progressing with a declined slope (as shown in the line graph). In fact, these symptoms disappeared completely and were represented by a median score of 0. On the other hand, symptoms such as cough and post-nasal discharge were persistent regardless of the surgical intervention. Bhattacharyya et al. [14] reported a significant reduction with a large effect size of all major CRS symptoms except hyposmia, which had a moderate effect size. However, the reported sequence of symptom improvement in that study started with facial pressure, nasal obstruction, congestion, rhinorrhea, and improvement in hyposmia [13]. DeConde et al. compared the improvement in CRS symptoms after medical treatment and with improvement after surgical treatment [15], and found that surgical management with ESS improves all major symptoms apart from olfaction three to four times more than medical treatment [16]. Many studies have indicated that the senses of smell and taste do not progress effectively after surgery; in fact, it can take up to 9.7 years on average to recover completely. This can be attributed to the location of the olfactory system, which is liable to be harmed either by the disease process or surgical injury [17]. A multi-institutional cohort study indicated that the percentage of patients who experienced a restored olfactory sense after surgery was relatively lower than of patients who did not [6].

As reported in previous literature, minor symptoms show a moderate effect change after surgery; fatigue however, shows a large effect change [13]. Our study results showed similar trends. Fatigue showed an achievable improvement, vanishing postoperatively to a median score of 0. The reduction in fatigue after surgery indicates that ESS not only affects the head and neck regions but also produces a systemic effect, indicating a considerable improvement in quality of life [13].

Researchers have asserted that cough is associated with less postoperative improvement. A plausible explanation for this is that the association of asthma or laryngopharyngeal reflux may induce cough regardless of the presence of CRS [4].

In the present study, a significant decline was noted in the ear and facial domain. This is in line with the findings of Stoikes et al. [18], which indicate that improvement in CRS symptoms following ESS is associated with the disturbance of normal mucociliary clearance in the nasopharynx tube. The mean preoperative global SNOT-22 score recorded in the present study was 40.3; this score decreased to 26 at the first postoperative visit and 22.18 at the last postoperative visit. Our results resemble the findings to the from the study by Hopkins et al., which was the first study that utilized the SNOT-22 score. In that study, the mean preoperative score was 41.7 and the postoperative score was 25.5 [9]. However, a second postoperative visit was not included in that study.

We analyzed each individual factor that may play a role in the outcome of ESS to achieve a controlled clinical steady state of CRS symptoms with better postoperative quality of life. This was most strongly demonstrated in the present study by history of AR. In fact, AR and CRS symptoms are a consequence of the inflammatory reaction that may lead to a special entity known as difficult-to-treat rhinosinusitis. Such factors associated with uncontrolled symptoms need to be identified and addressed to obtain an optimum control of the disease [19].

Tobacco use is known to affect outcomes, with poorer symptom scores and a higher prevalence of smoking observed amongst patients with refractory CRS. In the present study, 11.7% of the included patients were smokers, and they expressed poorer scores. Smoking is known to suppress sinonasal immunity, which results in bacterial stasis and biofilm formation that eventually worsens chronic rhinosinusitis [20]. For participants who were diagnosed with CRSwNP, the presence of nasal polyps is considered a contributing factor to the recurrence of nasal polyps 1 year after ESS, an outcome which has been estimated to occur in 20% of cases, and is known to adversely affect quality of life [21]. However, our analyses showed that CRS patients with concomitant asthma consistently had higher postoperative SNOT-22 scores, despite the fact that ESS has positive effects on asthmatic patients [22, 23].

This study examined the immediate and moderate postoperative effects of ESS, aiming to provide a more meticulous description of the improvement of each symptom and highlight how the selection of a more aggressive intervention can have a positive impact on patients’ symptoms and quality of life. Through investigating the detailed improvement of CRS symptoms following sinus surgery, the present study contributes to a better understanding of the underlying elements that influence physicians in selecting ESS as a treatment choice or an alternative option to medical therapy. With which these surgical intervention outcomes can be reasonably expected. Given the chronic nature of CRS, further investigation of the long-term improvement of each symptom using the SNOT-22 domain scores is recommended to facilitate proper planning of optimum management regimens.

Although this is a single center study which is considered as limitation in our design, our center is one of two governmental hospitals that provide ENT services of care in the region. In addition, the center is the only university teaching hospital in the area with an active ENT department; hence, we are considered a major referral hospital and receive patients from remote areas. Therefore, the results of this study might be extended beyond the registered patients in our hospital to reflect the population of the whole province.

We agree that the focus of this article is not novel; however, our study presents new findings regarding the role of allergy in the improvement of CRS [23]. This new finding, which has not been highlighted in the literature, might be due to unmeasured confounder effects (such as post-surgery medications) or the unique characteristics of our participants (such as Arabic ethnicity, which has had limited examination in the literature and might play a role in resolving the inflammatory process post-operatively). In addition, we provided a comprehensive description of the magnitude of differences and pattern of changes for the symptoms of CRS and its effect on the quality-of-life components, as measured by the SNOT-22 questionnaire at different time points post ESS.

## Conclusion

ESS helps in improving the symptoms of CRS to varying degrees and rates during the six-month period after surgery; this improvement varies significantly depending on the presence of a history of allergic rhinitis. Understanding the pattern of symptom improvement after ESS for CRS will aid patient counselling regarding selection of treatment modalities, expected outcomes, and associated predictive factors, to provide a strong incentive to optimize the current treatment protocols and maximize ESS outcomes and quality of life.

## Data Availability

The data that support the findings of this study are available from the corresponding author upon reasonable request.

## Abbreviations

CRS: Chronic rhinosinusitis
ESS: Endoscopic sinus surgery
SNOT-22: Sino-nasal Outcome Test-22
CRSwNP: Chronic rhinosinusitis with nasal polyposis
CRSsNP: Chronic rhinosinusitis without nasal polyposis
AR: Allergic rhinitis
